# Maximizing biodiesel production from waste cooking oil with lime-based zinc-doped CaO using response surface methodology

**DOI:** 10.1038/s41598-023-30961-w

**Published:** 2023-03-17

**Authors:** Mebrhit Gebreyohanes Weldeslase, Natei Ermias Benti, Mekonnen Abebayehu Desta, Yedilfana Setarge Mekonnen

**Affiliations:** 1 Center for Environmental Science, College of Natural and Computational Sciences, Addis Ababa University, P. O. Box 1176, Addis Ababa, Ethiopia; 2 Computational Data Science Program, College of Natural and Computational Sciences, Addis Ababa University, P. O. Box 1176, Addis Ababa, Ethiopia; 3 Department of Chemistry, College of Natural and Computational Sciences, Addis Ababa University, P. O. Box 1176, Addis Ababa, Ethiopia

**Keywords:** Environmental chemistry, Biodiesel

## Abstract

Biodiesel is one of the alternative fuels, commonly produced chemically from oil and methanol using a catalyst. This study aims to maximize biodiesel production from cheap and readily available sources of waste cooking oil (WCO) and lime-based Zinc-doped calcium oxide (Zn-CaO) catalyst prepared with a wet impregnation process. The Zn-CaO nanocatalyst was produced by adding 5% Zn into the calcinated limestone. The morphology, crystal size, and vibrational energies of CaO and Zn-CaO nanocatalysts were determined using SEM, XRD, and FT-IR spectroscopy techniques, respectively. The response surface methodology (RSM), which is based on the box-Behnken design, was used to optimize the key variables of the transesterification reaction. Results showed that when Zn was doped to lime-based CaO, the average crystalline size reduced from 21.14 to 12.51 nm, consequently, structural irregularity and surface area increased. The experimental parameters of methanol to oil molar ratio (14:1), catalyst loading (5% wt.), temperature (57.5 °C), and reaction time (120 min) led to the highest biodiesel conversion of 96.5%. The fuel characteristics of the generated biodiesel fulfilled the American (ASTM D6571) fuel standards. The study suggests the potential use of WCO and lime-based catalyst as efficient and low-cost raw materials for large-scale biodiesel production intended for versatile applications.

## Introduction

Energy is today a vital component of long-term economic development and maintaining a good quality of life for mankind. Even as fossil fuels contribute significantly to global energy demands, they are not renewable and their prices are unstable. Furthermore, the use of fossil fuels leads to the release of greenhouse gases (GHGs), which are responsible for the worsening of the world’s most serious environmental problems^1–7^. Carbon dioxide (CO_2_) emissions are predicted to rise to 40 thousand million kg by 2030. If the average global temperature rises by more than 2 °C in comparison to the pre-industrial era, up to one million species and hundreds of millions of people could become endangered^8^.

The interest in boosting renewable energy sources has increased in reaction to the depletion of fossil fuel supply and the realization that growing CO_2_ emissions worsen climate change^4,9,10^. Sustainable biofuel production is an important strategy for halting global warming, protecting biodiversity, boosting local economies, particularly in poor nations, and guaranteeing energy security worldwide^2,3^. Renewable and clean fuels derived from bioenergy feedstocks may assist in reducing global poverty, improving food security, accelerating economic development, and decreasing GHG emissions^11,12^. However, it is crucial to assess the real advantages of using biofuels over conventional energy sources using practical, scientific, and robust tools^13–16^. According to Astrup et al.^17^, life cycle assessment (LCA) has been identified as a thorough evaluation method for assessing the environmental impacts of the entire biodiesel production chain.

Nowadays, biodiesel is receiving a lot of interest because of its high biodegradability and low toxicity^18–21^. It can replace fossil fuels in many applications, such as internal combustion engines and transportation, without the need for significant retrofits. Moreover, it is believed that performance hasn’t changed much and that it releases almost no sulfates, aromatic compounds, or other hazardous chemicals. When considering the complete life cycle, CO_2_ emissions are minimal, and they seem to greatly increase the economic potential of rural areas^8^. Biodiesel is typically generated by the transesterification of renewable resources like animal fats or vegetable oils using methanol with an appropriate catalyst. Though biodiesel has numerous benefits compared to traditional diesel fuel, it has not been commercially successful in many countries, like Ethiopia, because of a shortage of appropriate feedstock. Since it raises conflicts between food and fuel, the use of animal fat and edible oils in the generation of biodiesel has drawn criticism. As a result, for its production, food-based feedstock must be replaced with low-quality non-edible feedstock^22,23^. In general, the life cycle assessment when converting biomass into biofuel as biodiesel is a complex process that requires careful consideration^13–16^. At the very beginning of the process, it is crucial to evaluate the environmental effects of the resources used for making biodiesel and costs associated with WCO collection, transportation, and pretreatment as well as costs incur during the transesterification process. Further elements need to be taken into account such as biomass conversion efficiency, air pollution due to emissions of gases from combustion, and impacts on environment and health. Moreover, issues like water consumption, waste to energy, and waste management aspects should be considered. Although higher yields are usually desirable for biodiesel production, this should not come at the expense of increased environmental impact if alternatives achieving similar yields with lesser environmental impact exist. Another key factor in the assessment is the carbon footprint of the entire process which must take into account all associated carbon intakes and outputs in comparison with the traditional fossil fuels^13–16^.

The high cost of the raw materials utilized in the synthesis of biodiesel is the primary barrier to its market competitiveness and commercial viability^24^. Biodiesel is more expensive than petrol-diesel. To address this issue, it is essential to work on alternative and less expensive feedstock and catalysts. The generation of biodiesel from waste, like WCO and CaO catalysts derived from limestone, is a viable option for producing cheap and environmentally friendly materials. WCO gathered from domestic cooking or cafés and restaurants may be used as a viable alternative for vegetable oil and animal fats because it can reduce total biodiesel generation prices while also addressing WCO disposal issues efficiently^25^.

Heterogeneous catalysts have attracted a lot of interest for use in the production of biodiesel due to their affordability, reusability, simplicity of catalyst separation, reduced corrosiveness, and low environmental impact^26^. Waste calcium carbonate-containing materials, like mollusk shells, eggshells, limestone, goat bone, and others, can be used to synthesize CaO^27,28^. CaO has recently gained significant attention and is frequently reported in the literature among heterogeneous catalysts due to its affordability, non-toxicity, and ease of availability^29^. However, the CaO catalyst that was on the market has significant drawbacks against the transesterification process, like moisture sensitivity and lower activity^30^. While it was reported that modified CaO was more active, it was unstable and leached Ca and/or active species into the reaction media.

ZnO is a common transition-metal oxide heterogeneous catalyst studied for the transesterification process^28^. The major disadvantage of ZnO-catalyzed transesterification reactions is the incomplete conversion of substrate to Fatty Acid Methyl Ester (FAMEs) (96.5%), though at high reaction temperatures. It has been reported that impregnation using K, Li, or Sr improves the ZnO activity^31^. Using a Li/ZnO catalyst and methanol to oil molar ratio of 20:1, an optimal conversion (96.3%) of soybean oil to matching methyl esters was achieved in 3 h. However, it has been shown that it is deactivated even after catalyzing the first reaction cycle due to Li leaching from the ZnO support^32^. Another author described the co-precipitation technique for producing CaO-ZnO by altering the Ca/Zn atomic ratio between 0.25 and 7.5% (wt.)^33^. After being rinsed with an NH_4_OH/CH_3_OH solution, the CaO-ZnO was reused up to three times as a catalyst (heterogeneous) for the transesterification of palm kernel oil, yielding 90% methyl ester concentrations^34^. The role that zinc plays in the production of biodiesel is the primary distinction between ZnO-CaO and Zn-CaO catalysts. In a ZnO-CaO catalyst, Zn functions as an acid to assist in transesterification, whereas in a Zn-CaO catalyst, Zn acts as an alkali to help in catalyzing the transesterification reaction. In general, transesterification reactions with Zn-CaO catalysts can result in relatively high yields of biodiesel but require longer reaction times than ZnO-CaO catalysts. Moreover, the biodiesel produced using ZnO-CaO as a catalyst exhibited incomplete conversion (96.5%), suffers from metal ion leaching, or needs a high methanol/oil molar ratio and high temperatures to achieve a sufficient FAME yield^35^. As far as we are aware, there is limited published work dealing with heterogeneous catalysts produced from Zn-doped CaO that is employed for triglyceride transesterification. More research is needed to find low-cost, long-term, and efficient feedstocks and catalysts for more practical biodiesel generation.

Therefore, the primary goal of this investigation is to improve the production of biodiesel from WCO feedstock by employing limestone-derived CaO and Zn-doped CaO nanoparticles and optimizing the main transesterification reaction parameters. The main objectives of this study were (1) prepare CaO nanocatalysts from limestone by calcination and increase their catalytic activity using zinc doping technique via the wet impregnation method (2) characterize CaO and Zn-CaO nanocatalysts using SEM, XRD, and FTIR techniques (3) produce biodiesel from WCO using a methanol and investigate the effect of transesterification reaction parameters such as temperature, reaction time, catalyst loading, and the molar ratio of methanol to oil using response surface methodology, and (4) examine the fuel characteristics test of produced biodiesel.

## Materials and methods

### Sample preparation of WCO

We obtained waste cooking sunflower oil from Addis Ababa residents that had been used to fry food. Filtration and dehydration methods were used in the pretreatment of WCO to get the removal of impurities. Filtration was used to remove food particles from the oil, and the water present in the WCO was then removed by heating the oil to 110 °C^22^. Standard procedures were used to measure the key physicochemical characteristics of WCO, including density, kinematic viscosity, ash content, molecular weight, acid, saponification, and free fatty acid (FFA) value, results are presented in Table 2.

### Preparation of nano-catalyst

#### Preparation of CaO nano-catalyst

The collected limestone was first washed repeatedly using distilled water to eliminate any contaminants. After that, it was dried overnight in a 120 °C oven. The limestone was then powdered using a grinder machine and passed across a 63 µm sieve mesh. Lastly, the powdered lime was calcined at 900 °C for 3 h before being stored in a desiccator for later use.

#### Zn-doped CaO nano-catalyst

Wet impregnation was used to synthesize a Zn-doped CaO nanocatalyst, which was then dissolved in double-distilled water for 4 h^28^. An aqueous solution of zinc sulfate dehydrate at the required concentration was added to this and stirred continuously for 6 h. Increasing Zn concentration can increase biodiesel conversion yield; however, it must be done carefully as too high a concentration could reduce reaction time and yields. Therefore, the concentration of zinc sulfate dehydrates was obtained at a Zn^2+^ concentration of 5% in the CaO, which was the ideal concentration for the production of biodiesel^36^. The resulting slurry was then filtered, and it was dried for 6 h in a 120 °C oven. The dried sample was then activated by being calcinated for 3 h at 800 °C and the catalysts generated were identified as 5% (wt.) Zn-CaO.

### Characterization of catalyst

The crystal structure and size of two different CaO samples were examined using XRD, one synthesized by calcining limestone at 900 °C and the other further improved by the wet impregnation method with zinc doping calcinated at 800 °C. Calcination at higher temperatures can cause sintering, which occurs when particles of a material are fused or heated together to form larger grains. This process can reduce the surface area of the catalyst, as smaller pores and pathways are lost due to the decrease in particle size and volume. Sintering also causes an increase in density, which generally reduces the catalytic activity of the catalyst due to decreased availability of active sites, hindering diffusional access of reactants. As a result, the optimum temperature should be chosen. Based on available literature, calcination temperatures of 900 °C and 800 °C were employed for CaO and Zn-CaO, respectively^18–20^. The XRD patterns of the samples were taken using a diffractometer employing Ni-filtered CuKα radiation at λ = 0.154 nm in the 2 theta 10–60° range. FTIR spectra were collected using a PerkinElmer spectrometer to detect changes in functional groups, and SEM images were taken using the FEI INSPECT 50 instrument to assess the morphology of the desired nano-catalysts. The origin software was used to analyze the XRD results, and the Debye Scherrer equation (D = Kλ/β cos θ) was used to determine the mean crystal size of the catalyst.

### Biodiesel production process and experimental design

The transesterification was conducted in a 500 ml round-bottomed flask with three necks and a magnetic stirrer. The reaction temperature is controlled by a thermometer in the side neck, while the center neck is used to enter a water-cooled condenser. WCO was taken and placed in a three-necked flask preheated to 50 °C for the reaction. After dissolving the catalyst in the required amount of preheated methanol, the calcium methoxide solution was added to the preheated oil. The transesterification process was then repeated to achieve the highest biodiesel yield possible by adjusting the operating parameters such as catalyst loading, alcohol-to-oil molar ratio, reaction time, and temperature. Centrifugation was used to isolate the solid catalyst once the reaction had been completed. The final product was then heated to remove any remaining methanol before settling in a separating funnel. Fatty acid methyl esters (biodiesel) make up the top phase, glycerol (a by-product) makes up the intermediate phase, and a catalyst makes up the bottom phase. The following equation was used to determine the biodiesel yield.

1 $$\mathrm{Biodiesel \; yield }\left(\text{\%}\right)=\frac{\mathrm{Biodiesel \; volume}}{\mathrm{volume \; of \; WCO}}\times 100$$

The Glycerol and biodiesel layer above the catalyst was then poured into another separating funnel, with the catalyst remaining at the bottom. After two hours, the glycerol and biodiesel layers could be differentiated once again. The separating funnel’s knob was then opened, and glycerol biodiesel was collected in the flask.

### Biodiesel production using response surface methodology (RSM)

The statistical software Minitab (Version 10.0.6, Stat-EaseInc., MN, USA) and RSM based on Box-Behnken design (BBD) were used to examine the maximization of operating parameters in the transesterification reaction. To maximize biodiesel conversion (Y), four independent variables, including methanol to oil ratio (g/g), reaction time (min), temperature (°C), and catalyst loading (% wt.) at three levels (− 1, 0, 1), were examined. The ratio of methanol to oil, reaction time, temperature, and catalyst loading are denoted, respectively, by the letters A, B, C, and D. Table 1 depicts the range and values of each independent variable utilized in this investigation. The experimental setup includes 27 runs, which are computed using the formula: 2^n^ + 2n + n_c_, where n represents the total number of independent variables (n = 4), 2n represents the number of axial points, 2^n^ represents the number of factorial points, and n_c_ represents the number of replicated central point^37^. As a result, the software developed for this investigation includes three central points with one block, eight axial points, and six factorial points.

**Table 1:** The RSM design’s independent variables and their levels.

Independent variables	Unit	Symbol	Levels		
			Low (− 1)	Center (0)	High (1)
Methanol to oil ratio	g/g	A	6:1	10:1	14:1
Reaction time	min	B	60	120	180
Reaction temperature	°C	C	50	57.5	65
Catalyst loading	wt.%	D	1	2.5	5

### Statistical data analysis

MINITAB software was used to analyze the experimental results in RSM. A mathematical model was designed to determine the functional relationship between the response and the independent variables. A second-order polynomial equation is employed in the mathematical model (Eq. (2)) to explain the influence of variables on linear, quadratic, and interaction terms^38^.

2 $$\mathrm{Y}= {\mathrm{b}}_{\mathrm{o }}+ \sum_{\mathrm{i}=1}^{\mathrm{k}}{\mathrm{b}}_{\mathrm{i}}{\mathrm{X}}_{\mathrm{i }}+ \sum_{\mathrm{i}=1}^{\mathrm{k}}{\mathrm{b}}_{\mathrm{ii}}{\mathrm{X}}_{\mathrm{i}}^{2} + \sum_{\mathrm{i}<\mathrm{j}}^{\mathrm{k}}{\mathrm{b}}_{\mathrm{ij}}{\mathrm{X}}_{\mathrm{i}}{\mathrm{X}}_{\mathrm{j}} +\dots +\mathrm{e}$$

where Y denotes the response variable, the intercept, linear coefficient, interaction effect, and quadratic coefficients are denoted by b_0_, b_i_, b_ij_, and b_ii_, respectively, and the random error by e. This equation depicts the empirical relationship between the output and the independent parameter as obtained by Box-Behnken RSM modeling.

The statistical validity of the suggested model and model terms was assessed using ANOVA. The coefficients of determination (R^2^) were employed to assess the model’s quality. The P-value and a 95% confidence level were utilized to evaluate the importance of the model terms. To assess the relationship between the independent variables and their responses, contour and surface plots were developed.

## Results and discussion

### Characteristics of WCO

The main physicochemical properties of WCO, including density, acid value, free fatty acid value, saponification value, kinematic viscosity, and molecular weight were measured after filtration and dehydration. The results of the physicochemical properties are shown in Table 2.

**Table 2:** The main physicochemical characteristics of sunflower WCO.

Properties	Unit	Obtained value of WCO	Edible sunflower oil	
Density@20 ℃, g/ml	g/ml	0.9156	1.069	^39^
Acid value	mg KOH/g oil	2.01	< 0.6	WHO^40^
Free fatty acid (FFA) value	%	1.005	0.14	
Kinematic viscosity at 40 ℃	mm^2^/s	46.37	1.5–6	WHO
Saponification value	mg KOH/g oil	213.6475	21.09	^41^
Molecular weight	g/mol	785.736	877	^42^

### Characterizations of prepared lime-based nanocatalyst

#### Particle size analysis

**Figure 1:**
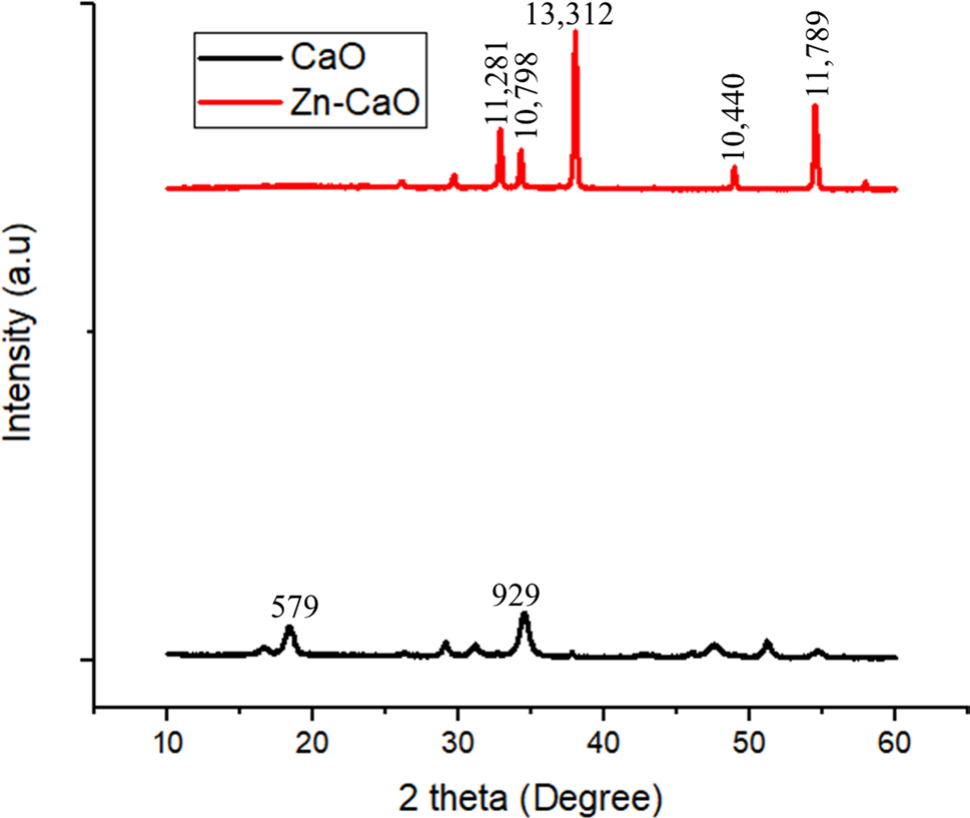
The XRD results of lime-based pristine CaO and Zn-doped CaO (5% wt.) nanocatalyst.

Figure 1 depicts the XRD pattern for CaO formed by calcinating limestone at a temperature of 900 °C displayed sharp peaks at 2-theta (2θ) of 17.86°, 29.12°, 34.24°, 48.48°, and 51.64°. Similar high-intensity 2θ peak values were observed for Zn-CaO synthesized by calcination at 900 °C followed by wet impregnation at 30.31°, 34.36°, 38.1°, 48.88°, and 54.72°. The XRD results agree well with a previous report on limestone-derived CaO powder^29^. The observed sharp peaks in the XRD pattern indicate the synthesis of crystalline nanocatalysts. The Debye Scherrer equation was employed to determine the average crystallite size using XRD peak width analysis. Accordingly, the average crystallite sizes of lime-based CaO nanoparticles before and after zinc doping were found to be 21.14 nm and 12.51 nm, respectively. Hence, the latter catalyst, Zn-CaO, has a smaller average particle size, which improves surface area and hence enhances the catalytic performance of the transesterification reaction. The primary cause of the reduction in crystallite size is the foreign Zn^2+^ impurities, which disturb the host CaO lattice.

#### Fourier transforms infrared spectroscopy (FTIR)

**Figure 2:**
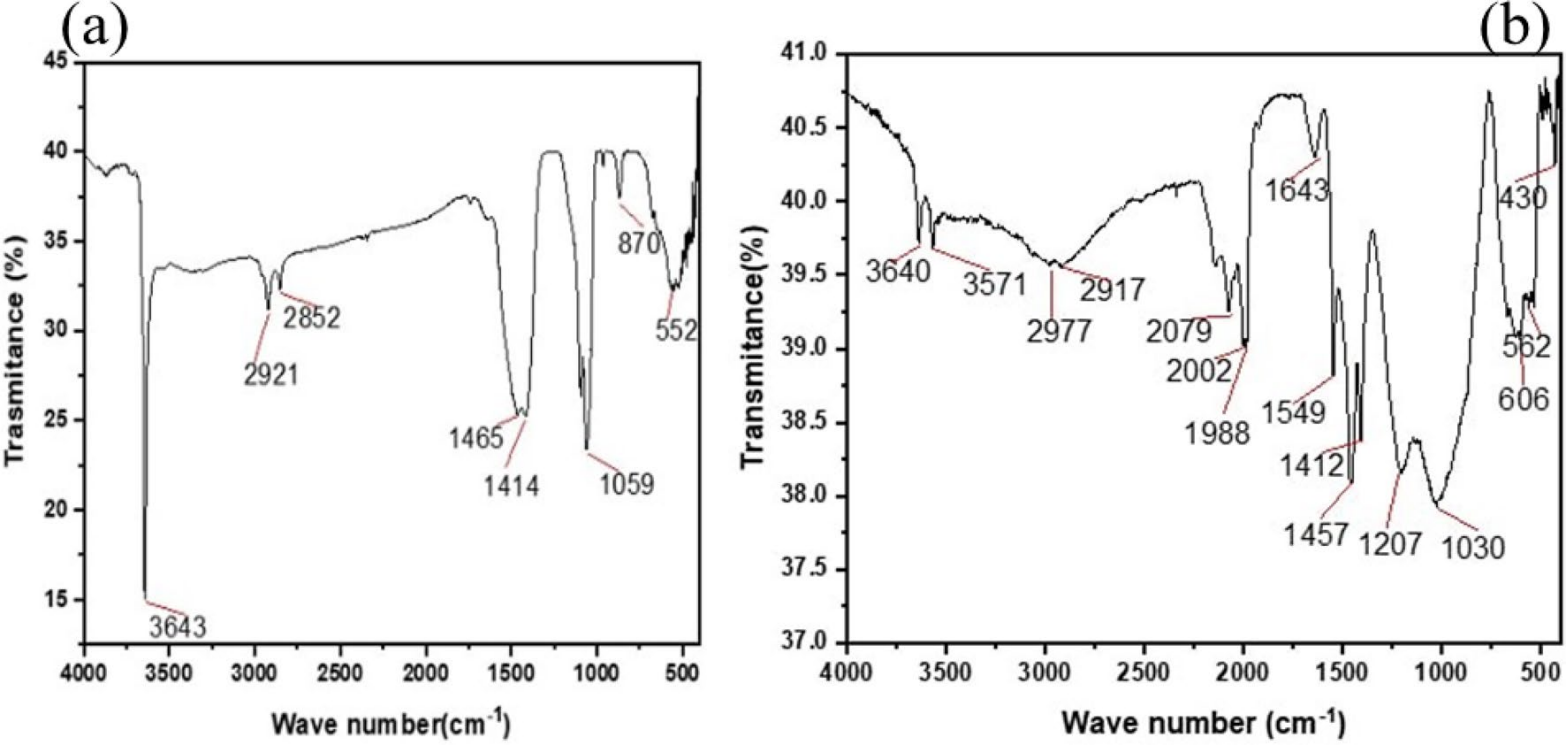
FTIR Spectrum of limestone pattern of (**a**) CaO and (**b**) Zn-doped CaO nanocatalyst.

Figure 2 shows the IR spectra of the lime-based pristine CaO and the Zn-doped CaO nanocatalyst. The sharp absorption band sat 3643 cm^−1^ arise due to the stretching mode of the OH group bonded to the metal ions, indicating the existence of small amount of water absorbed by the CaO nanocatalyst (Fig. 2a) and this peak turn out to be very weak in the Zn-CaO nanoparticles (Fig. 2b). High energy bands at 1465, 1414, 1059, 870, and 552 cm^−1^ were observed for the CaO nanocatalyst, while Zn doping onto the CaO shifts the characteristic peaks to 1457, 1412, 1207, 1030, 606, and 562 cm^−1^. For Zn-CaO, a weak peak at 1988–2079 cm^−1^ is associated with the carbonate C=O bond. The broad band at 1400–1550 cm^−1^ can be associated with the asymmetric stretching vibration of (C–O) related to the carbonation of CaO. The characteristic stretching mode of Ca–O is assigned to the significant band sat 552 cm^−1^ and this peak ultimately split into two higher wave number bands, 562 and 606 cm^-1^, due to the Zn–O vibrations (which replace some of the Ca–O bonds) and the out of a plane and in of plane Vibrational Modes of (C–O) related to carbonation. Generally, the FTIR data shows zinc doping to lime-based CaO facilitates the liberation of carbonate metal and the formation of Zn-CaO with broader and more intense peaks upon the addition of Zn.

#### Morphology analysis

**Figure 3:**
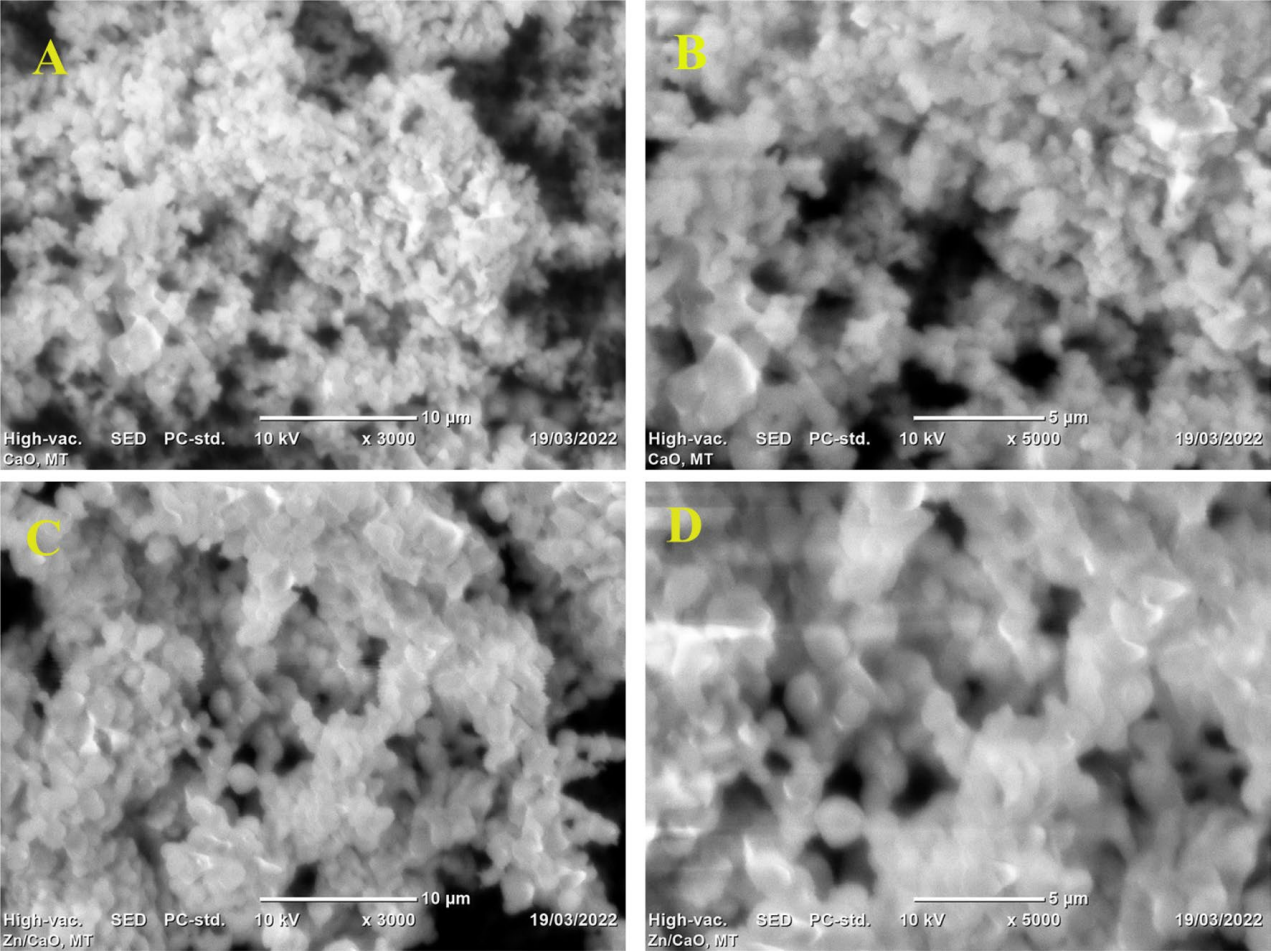
SEM images of lime-based CaO (**A**,**B**) and 5% Zn-doped lime-based CaO (**C**,**D**) taken at 10 and 5 µm, respectively.

The SEM was employed to determine the morphology of the limestone-based CaO powder before and after doping with Zn. Micrographs were taken at magnifications of 10 and 5 µm. As shown in Fig. 3, the Zn-doped CaO nanocatalyst calcinated at 900 °C for 3 h exhibited a larger grain surface compared to undoped CaO. According to the SEM images, the Zn-doped lime CaO generally has irregularly shaped particles, a porous structure, and a high number of active sites. In other words, the particles varied in size, distribution, and form, indicating that the later nanocatalyst ensured a higher surface area for the transesterification reaction. The significant clustering of the Zn additive with the CaO during catalyst synthesis and heating at high temperature may be responsible for the observed difference in form and morphology^35^.

### Effect of key transesterification parameters

#### Regression model development

The RSM was applied to optimize the four key transesterification variables namely, methanol to oil molar ratio, temperature, catalyst loading, and reaction time across a total of 27 experiments. Table 3 displays the experimental design as well as the actual and predicted FAME content values. At a temperature of 57.5 °C, methanol to oil molar ratio of 56.41 g/g, for 120 min, and with 5% (wt.) of catalyst loading, the highest production of FAME was gained. However, the lowest FAME yield was achieved at 24.18 g/g methanol to oil ratio, 120 min of reaction time, 57.5 °C temperature, and 1% (wt.) catalyst loading. Towards predicting the FAME yield, the data obtained from experiments were subjected to various nonlinear regression analyses, which produced a quadratic model (Eq. (3)).

3 $${\text{Y}} = { 121 } + { 2}.{\text{85 A }} + \, 0.00{\text{3 B }} - { 5}.{\text{61 C }} + { 12}.{\text{92 D }} - \, 0.0{\text{2137 A}}^{{2}} + \, 0.000{\text{257 B}}^{{2}} + \, 0.0{\text{492 C}}^{{2}} - \, 0.{\text{444 D}}^{{2}} + \, 0.00{\text{241 A}}*{\text{B }} + \, 0.00{\text{12 A}}*{\text{C }} - \, 0.0{\text{973 A}}*{\text{D }} - \, 0.00{1}0{\text{6 B}}*{\text{C }} - \, 0.0{\text{175 B}}*{\text{D }} + \, 0.0{\text{37 C}}*{\text{D}},$$

where Y refers to the predicted responses and letters A, B, C, and D represent the code values given for the test variables methanol to oil, temperature, reaction time, and catalyst load, respectively. AB, AC, AC, BC, BD, and CD refer to the interaction terms, while A^2^, B^2^, C^2^, and D^2^ are the quadratic terms. A positive parameter in a regression equation indicates a synergistic impact in which the result grows as the input of independent variables increases. A negative sign, on the other hand, implies a contrasting effect wherein response increases with decreases in input factors^43^.

**Table 3:** Key transesterification reaction parameter optimization with the RSM.

Run	A. methanol/oil (g/g)	B. time (min)	C. temp. (°C)	D. catalyst (wt.%)	Experimental FAME %	Predicted FAME %
1	24.18	60	57.5	3	51.65	51.4358
2	56.41	60	57.5	3	85.1	85.3875
3	24.18	180	57.5	3	52	52.5308
4	56.41	180	57.5	3	92.78	95.8125
5	40.295	120	50	1	60.6	64.1725
6	40.295	120	65	1	60.9	64.3042
7	40.295	120	50	5	91	88.4142
8	40.295	120	65	5	92.5	90.7458
9	24.18	120	57.5	1	38	33.4737
10	56.41	120	57.5	1	81.2	78.3654
11	24.18	120	57.5	5	66	65.0904
12	56.41	120	57.5	5	96.65	97.4321
13	40.295	60	50	3	75	75.6371
14	40.295	180	50	3	87.42	82.3521
15	40.295	60	65	3	76.5	77.8237
16	40.295	180	65	3	87.61	82.6287
17	24.18	120	50	3	50.04	53.3587
18	56.41	120	50	3	91.56	91.6854
19	24.18	120	65	3	52.5	54.3004
20	56.41	120	65	3	94.6	93.2071
21	40.295	60	57.5	1	59.2	57.4121
22	40.295	180	57.5	1	66.2	67.3721
23	40.295	60	57.5	5	86.2	86.9537
24	40.295	180	57.5	5	87.8	88.5137
25	40.295	120	57.5	3	75.85	75.9167
26	40.295	120	57.5	3	75.6	75.9167
27	40.295	120	57.5	3	76.3	75.9167

#### Statistical analysis and diagnostic of model adequacy

The ANOVA findings are displayed in Table 4. The F value of 94.37 shows that the model is highly relevant, and model terms are significant when the “Prob > F” value is less than 0.05. According to Anwar (2018), more significant coefficients have a higher F-value and a lower P-value^36^. The conversion of biodiesel was severely impacted (P < 0.05) by three linear terms (A, B, and D), five interactive terms (AB, AC, AD, BD, and CD), and one quadratic term (A^2^), while the other terms of the model had no significant effect (P > 0.05) on biodiesel production. The F- and P- values for lack of fit were 132.84 and 0.211, respectively, indicating that the lack of fit was not statistically significant when compared to the pure error. The model fit was appropriate (Vasaki et al.^47^). The coefficients of determination (R^2^-values) are used to assess the quadratic model’s goodness of fit^44^.

**Table 4:** ANOVA of the proposed model for FAME production.

Source	Degree of freedom (DF)	Sum of squares	Mean square adj MS	F-value	P-value	Remark
Model	14	6907.61	493.40	94.37	0.000	Significant
Linear	1	6504.42	1626.11	116.56	0.000	
Methanol/oil	1	4473.74	4473.74	320.68	0.000	
Time	1	99.53	99.53	7.13	0.020	
Temperature	1	4.55	4.55	0.33	0.005	
Catalyst	1	1926.60	1926.60	138.10	0.000	
Square	1	322.20	80.55	5.77	0.008	
A^2^	1	164.21	164.21	11.77	0.005	
B^2^	1	4.55	4.55	0.33	0.578	
C^2^	1	40.92	40.92	2.93	0.112	
D^2^	1	16.85	16.85	1.21	0.293	
2-way interaction	1	80.98	13.50	0.97	0.0486	
AB	1	21.76	21.76	1.56	0.0235	
AC	1	0.08	0.08	0.01	0.0139	
AD	1	39.38	39.38	2.82	0.0119	
BC	1	0.91	0.91	0.07	0.0803	
BD	1	17.64	17.64	1.26	0.0283	
CD	1	1.21	1.21	0.09	0.0473	
Error	1	167.41	13.95			
Lack-of-fit	10	167.16	16.72	132.84	0.211	Not significant
Pure error	2	0.25	0.13			
Total	44	20,961.96				

R^2^ = 0.9763, R^2^ (adjusted) = 0.9754, R^2^ (predicted) = 0.9539, adequate precision = 54.58, CV = 2.05%.

**Figure 4:**
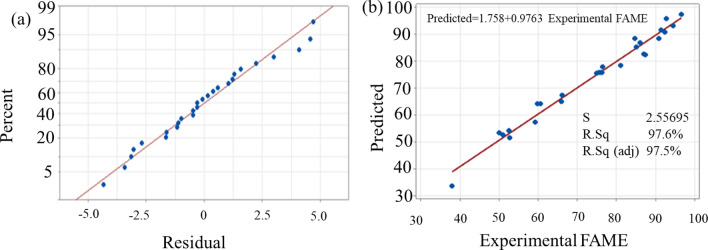
(**a**) Normal probability plot (response is Experimental FAME) and (**b**) FAME conversion plot of actual vs. predicted.

The obtained values for predicted R^2^, correlation coefficient R^2^, and adjusted R^2^ were 0.9539, 0.9763, and 0.9754, respectively. The higher the R^2^ value, the higher the model’s reliability in predicting biodiesel conversion^45^; the adjusted R^2^ evaluated the amount of variance around a mean described by the model^46^. According to this study’s R^2^-value, the quadratic model accounted for 97.63% of the variability in biodiesel output. The high adjusted R^2^ value implied good agreement between the observed and predicted biodiesel yield values, suggesting that the intended quadratic model equation produces good and accurate results. Moreover, a high level of agreement is indicated by the fact that the difference between the projected and adjusted R^2^ is too small^47^. The observed and predicted biodiesel production levels are highly correlated, as indicated by R^2^ and adjusted R^2^ values close to one. Similarly, the model’s low coefficient of variance (2.05%) suggested that the experimental data was accurate and reliable^48^. As a result, the proposed model predicted biodiesel production across a broad range of experimental parameters. The proposed model’s suitability was further assessed using diagnostic plots like predicted vs. actual and normal probability plots (Fig. 4)^49^. Figure 4b depicts a plot of predicted vs. actual biodiesel conversion value. The results were revealed to be closer to a straight line, implying that the predicted results generated by the designed model corresponded satisfactorily with the experimental results. A normal probability plot of the residuals for biodiesel conversion is shown in Fig. 4a, demonstrating that the errors were normally distributed in a straight line. This implies that the residuals of biodiesel conversion followed a typical distribution^50^.

### Combined effects of variables on biodiesel conversion

**Figure 5:**
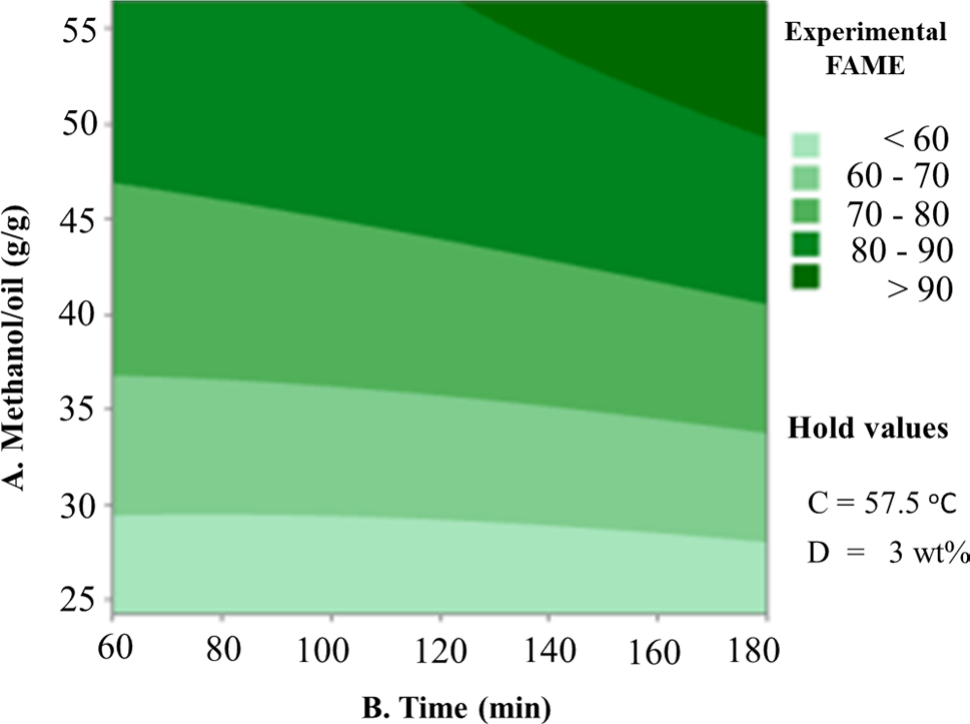
Contour plot of experimental fame vs. methanol to oil (A), Time (B).

**Figure 6:**
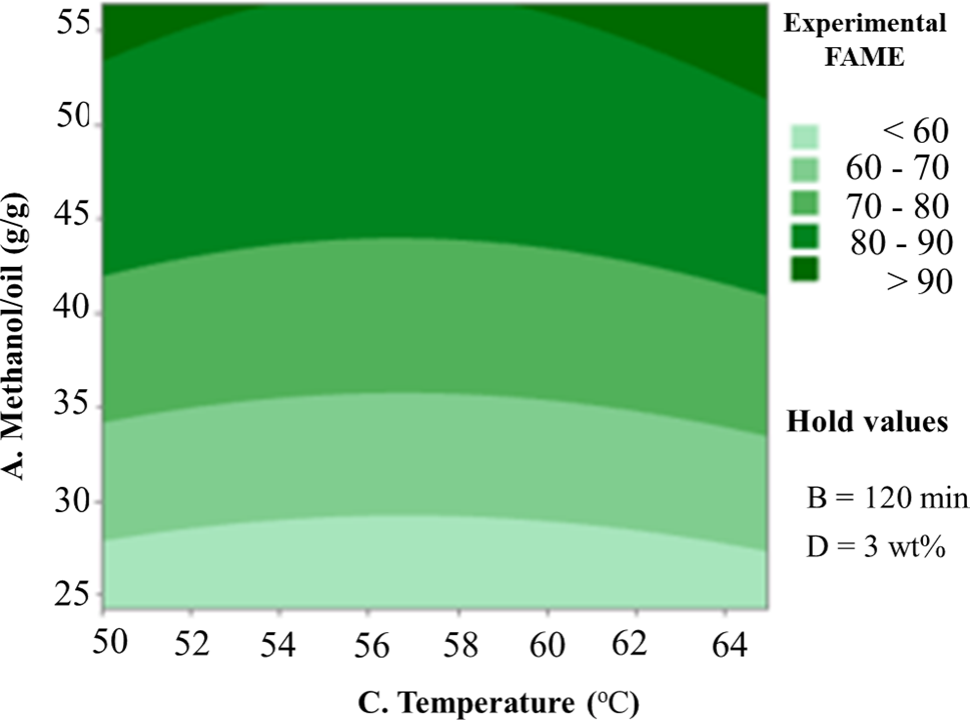
Contour plot experimental fame vs. (A) Methanol to oil (C) Temperature.

**Figure 7:**
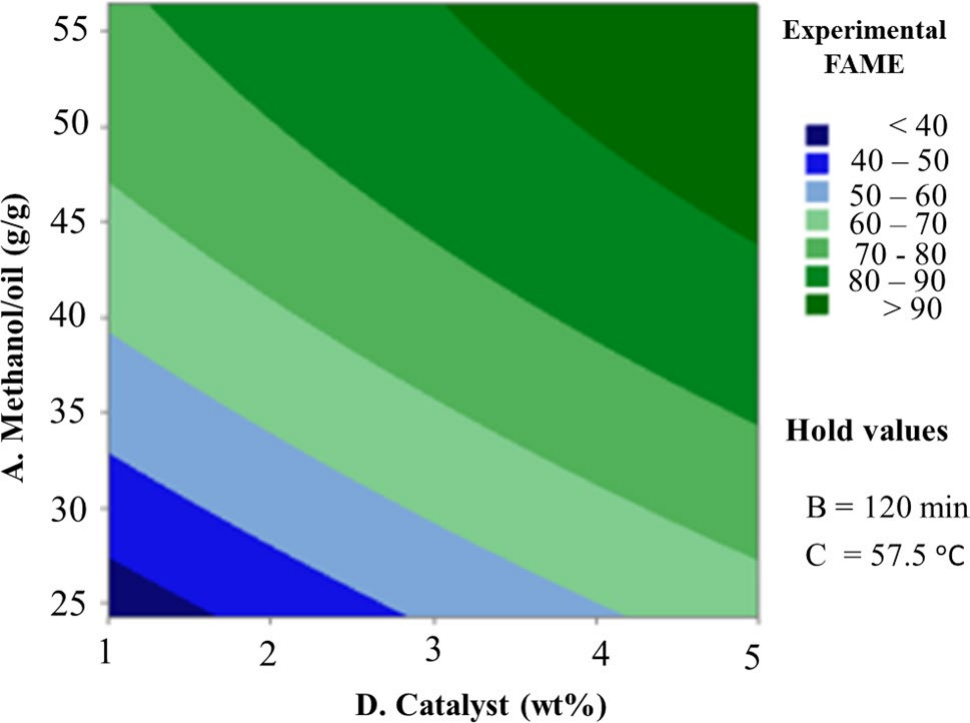
Contour plot of experimental FAME vs. (A) Methanol/oil (D) Catalyst.

**Figure 8:**
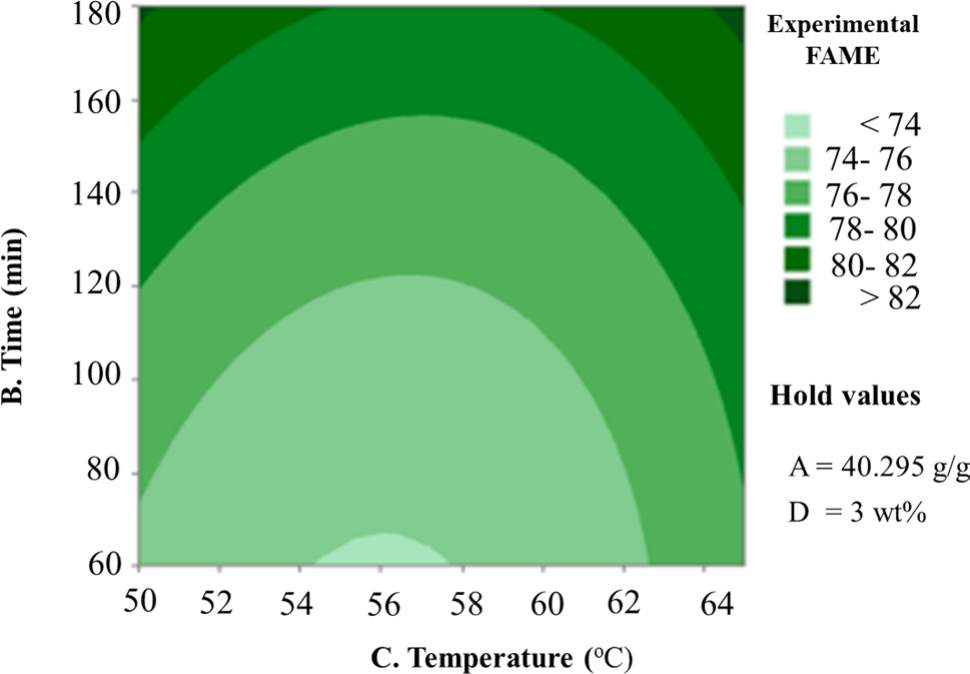
Contour plot experimental fame vs. (B) Time (C) Temperature.

**Figure 9:**
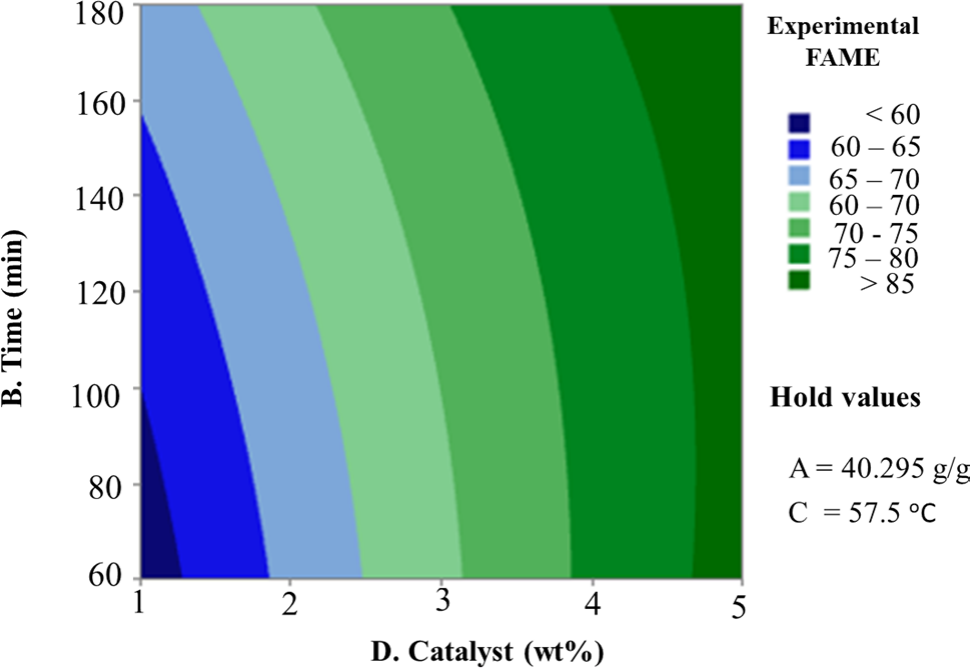
Contour plot of experimental FAME vs. (B) Time (D) Catalyst.

**Figure 10:**
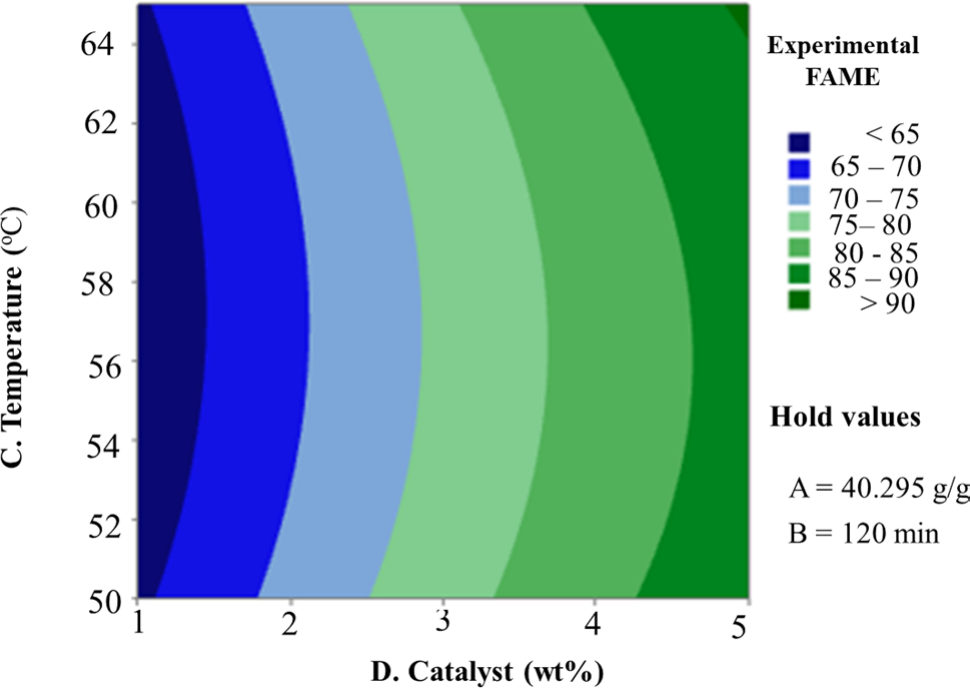
Contour plot of experimental FAME vs. (C) Temperature (D) Catalyst.

2D contour plots were utilized to examine the concurrent effect of the independent factors on the production of biodiesel. The quadratic model equation was used to create these plots in order to understand how process variables affect the conversion of biodiesel. The plots show how two variables interact while keeping the other two constant or fixed. They were used to obtain the best possible condition for each independent variable to maximize biodiesel conversion. Figures 5, 6, 7, 8, 9 and 10 illustrate a contour plot of the interactions of the methanol-to-oil ratio, temperature, reaction time, and amount of catalyst in the biodiesel-making process. Figure 5 depicts the impact of methanol to oil ratio and reaction time on biodiesel production at constant temperature and amount of catalyst. The methanol-to-oil ratio and reaction time both affect how much biodiesel is converted, but the increment that results from an increase in reaction time is less noticeable, as indicated by the linear slope. Conversion, on the other hand, increases sharply with increasing methanol to oil ratio from 51.65 to 92.78% of yield in the range of 24.18 methanol ratio with respect to 60 min reaction time to 56.41 methanol to oil ratio with respect to 180 min reaction time (Fig. 5). The most significant parameter in this model is the methanol-to-oil ratio. Since transesterification is a reversible reaction, sufficient methanol is needed to drive the process in the direction of the final product, the biodiesel^22^. The optimum biodiesel conversion of 96.65% was obtained with a 5-Zn-CaO nanocatalyst under optimum reaction conditions methanol to oil molar ratio of 14:1, a temperature of 57.5 °C, catalyst loading of 5% (wt.), and reaction time of 120 min.

Figure 6 shows the couture plot of the combined influence of reaction temperature and methanol-to-oil ratio. The temperature has a significant impact on reaction speed, resulting in increased ester conversion. The yields of biodiesel increase dramatically, moving from 50 to 94.6%, when the reaction temperature is increased from 50 to 65 °C and the methanol to oil molar ratio is increased from 24.18 to 56.46. Because of the subcritical condition of methanol at low temperatures, relatively low conversion to methyl ester is observable. Methanol evaporates at temperatures greater than its boiling point, reducing the production^36^. In addition, the methanol-to-oil ratio had a major impact on yield, which reduced substantially the high value of those. The OH group in the alcohol interacts with triglycerides to trigger hydrolysis and the production of soap as a molar ratio of methanol to oil rises. The interaction of these factors is of relevance since the optimal temperature variation of the methanol-to-oil ratio influences the reaction yield significantly^25^.

Figure 7 shows how catalyst loading and the methanol-to-oil ratio interact to affect the biodiesel yield at constant temperature and reaction time. The graph clearly illustrates that both catalyst loading and methanol to oil ratio boost the conversion of biodiesel production. The methanol to oil ratio, however, is the most important variable in this case, as evidenced by the very massive rise in conversion with rising methanol to oil ratio from 38 to 96.65% yield in the range of 24.18 methanol to oil ratio with respect to 1% (wt.) up to 56.41 methanol to oil ratio with respect to 5% (wt.) catalysts, respectively. This might be because increasing methanol content shifts the balance to the product side, promoting the biodiesel production^51^. The catalyst concentration enhanced biodiesel production, but rising the methanol-to-oil ratio above the optimum level reduced the biodiesel yield^52^.

The couture plot of the paired influence of temperature and time is shown in Fig. 8. It has been found that biodiesel conversion increases with reaction time, which is due to raising the reaction temperature to the alcohol boiling point. However, increasing the reaction temperature might induce methanol loss and, as a result, slow down the process, resulting in increased FFA of the esterified oil, and a comparable reason was accounted for^36^. The interaction between reactants increases with increasing time and temperature, resulting in an increase in conversion from 75 to 87.61% in the range of reaction temperature 50 °C versus 60 min reaction time up to reaction temperature 65 °C versus 180 min reaction time (Fig. 8). The ideal temperature for oil methanolysis is shown by several earlier studies to be 65 °C, which is the boiling point of methanol at atmospheric pressure. Higher temperatures than the optimal do not reduce response time or enhance the conversion rate^28^. The production of biodiesel is reduced when the reaction temperature rises above the boiling point of methanol because it begins to evaporate^53^.

Figure 9 depicts how catalyst loading and reaction time interact during the conversion of biodiesel. Catalyst loading has been discovered to have a bigger effect on biodiesel conversion than reaction time. Based on the very sharp slope of the catalyst loading, which ranges from 59.2 to 87.8% in the time reaction range of 60 min with respect to catalyst load of 1% (wt.) up to time reaction 180 min with respect to catalyst load of 5% (wt.), increasing time reaction is helpful to convincing of production by increasing the catalyst’s surface to regenerate the active sites^38^. FAME yield increases when catalyst concentration is increased, reaching a maximum of 96.65%. It was observed that raising the catalyst concentration in the process from 0.91 to 4.5775 g considerably boosted the biodiesel yields from 59.2 to 87.8%. The abundance of active sites accessible for the surface reaction and the unrestricted mass transfer, which is a characteristic of the employment of these catalytic systems, are related to this behavior. However, it was shown that a greater catalyst concentration showed a reduction in the production of FAME, which may be attributable to the restrictions placed by the reaction’s equilibrium^36^.

Figure 10 displays the 2D counterplots of the interaction effects of catalyst concentration and reaction temperature. Biodiesel production rises when temperature and catalyst parameters are changed. The greater yield of biodiesel is 92.5%, obtained at 65 °C, and the temperature rise is extremely significant. This is due to the triglyceride molecules’ carboxylic group being rapidly activated at high temperatures and being accessible for nucleophilic attack by the hydroxyl group of the CH_3_OH^54^. Triglyceride activation is generally challenging because these molecules’ potential long alkyl chains may interfere. The temperature needs to help activate the carbonyl groups in the contraindicate constants in order to boost the nucleophilic attack of methanol on the triglyceride molecules. Because of the denaturation of free enzymes, a temperature rise reduces the yield of the biodiesel^55^. Furthermore, when reactants are miscible, high temperatures accelerate the diffusion rate, enabling faster reaction rates across a wider temperature range, which could assist in maintaining the catalytic activity. Once the reaction temperature is reached, the yield of biodiesel decreases due to the polarity of the methanol, which reduces the concentration of methoxide particles in the reaction mixture and, consequently, the activity of the catalyst surface. It is also possible that saponification phenomena and improper mixing of the solution mixture with extra catalyst used during the transesterification reaction are to blame^56^.

### Characteristics of produced biodiesel

As shown in Table 5, key fuel properties of the generated biodiesel such as density, flashpoint, kinematic viscosity, acid value, cloud point, water and sediments, copper strip corrosion, and calorific value were examined as per the American fuel standards of ASTM D6751 and EPSE limits. The overall analysis results showed that the properties of biodiesel produced fell within the acceptable standard as shown in Table 5 and in good agreement with the previously reported related works^25,57^.

**Table 5:** Physicochemical characteristics of the biodiesel produced from WCO.

No	Property	Units	Test method ASTM	ASTM limit for B 100	Biodiesel produced
1	Density @15 °C	kg/m^3^	D4052	880	869
2	Density @20 °C	kg/m^3^	D4052	880	860
3	Kinematic viscosity at 40 °C	mm^2^/s	D445	1.9 $-6$	3.56
4	Cloud point °C		D2500	− 3 to − 15	11
5	Acid value	mg KOH/g	D974	0.50	0.43
6	Flash point °C		D93	100 $-170$	130
7	Water and sediment	% v/v	D2709	0.050	0.030
8	Copper strip corrosion, 3 h @100 °C		D130	3 max	1.5
9	Calorific value	BTU/LB	Calculated	–	32,764.80
10	Color		Observed		Light yellow

Table 6 presents a comparison of related previous studies conducted using various optimization techniques such as Design of Experiments (DOE), Response Surface Methodology (RSM), and Machine Learning algorithms (ML). The findings of this study demonstrate that the approach taken was accurate, and the output was higher than that of most other studies.

**Table 6:** A comparison of related works with this work in terms biodiesel yield, optimization technic and optimization parameters.

Feedstock	Optimization methods		Alcohol: oil ratio	Catalyst loading	Temperature and reaction time	Yield (%)	References
Waste date seed oil	RSM		15:1	1.5% (wt.), magnetic solid acid	55 °C for 47 min	91.4	^13^
WCO	ML	T2FLS	10:1	1.5% (wt.) CaO derived from Ostrich egg shells	65 °C for 90 min	96.7	^58^
		T1FLS				96.8	
		ANFIS				96.4	
	RMS					97.5	
WCO	DOE		12:1	2.5% (wt.) CaO (eggshell)	60 °C for 2 h	94	^20^
WCO	DOE		8:1	1% (wt.) CaO from Ca(NO_3_)_2_.4H_2_O	50 to 55 °C for 90 min	96	^18^
Scenedesmus algal	DOE		11:1	1% (wt.) CaO (waste)	60 °C for 3 h	92	^19^
WCO	RSM		14:1	5% (wt.) Zn-CaO	57.5 °C for 2 h	96.65	This work

## Conclusion and future work

In this investigation, biodiesel was generated from readily available waste cooking sunflower oil utilizing low-cost lime-based Zn-CaO nanocatalyst designed by wet impregnation followed by calcination at 900 °C. Optimization of 27 runs from four key parameters was made using RSM based on the box-Behnken design. The synthesized Zn-CaO exhibited irregularly shaped particles, a porous structure, a significant number of active sites, and lower particle size indicating that the Zn-doped catalyst had a larger surface area for transesterification reaction compared to pristine CaO, consequently maximizing the catalytic activity of the biodiesel production. Furthermore, the XRD patterns revealed that the average crystallite sizes for pristine CaO and Zn-CaO nanoparticles calcined at 900 °C followed by the wet-impregnated technique were 21.14 nm and 12.51 nm, respectively. As a result, the Zn-doped CaO proved superior catalytic performance compared to pristine CaO, with an optimum biodiesel yield of 96.65% obtained at experimental conditions of 57.5 °C reaction temperature, 5% (wt.) catalyst loading, 14:1 methanol to oil molar ratio, and 2 h of reaction time, with the result being nearly the same as the RSM predicted yields, validating the model’s accuracy. The outputs of the RSM-based optimization show the significance of the proposed models and that the predictions well fit to the experimental data. The statistical study also revealed that all four parameters had a substantial impact on the efficiency of biodiesel production. The fuel properties of the produced biodiesel were examined as per the American ASTM standards and found to meet the standards. Therefore, it can be concluded that low-cost lime-based Zn-doped CaO catalysts, could be used to design and develop economical and large-scale biodiesel production from WCO.

Further research on the following subjects is possible (a) Investigate effective ways to measure and reduce greenhouse gas emissions associated with the production of biodiesel from WCO by looking at potential resources, process control strategies, and feedstock components and modifications (i.e. deoxygenation). (b) Assessing potential waste cooking oil resources, designing efficient collection mechanisms, estimating the cost of collecting and transporting waste cooking oil to production sites, and planning pretreatment methods before use are crucial steps in the development of economically viable, environmentally benign, and cost-effective large-scale biodiesel production. (c) LCA can also be an important tool in gaining a better understanding of environmental implications associated with biodiesel production. LCA evaluates key environmental impacts such as energy use, greenhouse gas emissions and other environmental pollutants, from raw material extraction to final product disposal^13^. (d) Heat and mass transfer limitations can become a significant challenge when scaling up biodiesel production from the lab scale to the commercial scale. Future research should focus on making biodiesel production more cost-effective on a large-scale basis. This would include studying conditions for reducing waste streams, optimizing reaction times, developing better catalysts and alcohol for use in the production process, employing techniques such as micro channeling to improve heat and mass transfer performance, and ways to incorporate renewable energy sources like solar energy into the process^59^. (e) Blending renewable materials with diesel offers the potential for improved engine performance and reduced smoke emissions^15^. Future research should focus on exploring various processing methods to maximize the efficiencies of blending, as well as analyzing data collected from experiments conducted on blends of biodiesel and renewable materials. Researchers should also be looking into ways to increase production yields while maintaining optimal combustion efficiency. Additionally, further studies could be carried out to explore fuel properties at different temperatures and environments, in order to determine the most effective combinations for long-term sustainability.

## Data Availability

All data generated or analysed during this study are included in this published article.
